# Association of cellular and molecular responses in the rat mammary gland to 17β-estradiol with susceptibility to mammary cancer

**DOI:** 10.1186/1471-2407-13-573

**Published:** 2013-12-05

**Authors:** Lina Ding, Yang Zhao, Christopher L Warren, Ruth Sullivan, Kevin W Eliceiri, James D Shull

**Affiliations:** 1 McArdle Laboratory for Cancer Research, Department of Oncology, School of Medicine and Public Health, University of Wisconsin Madison, 1400 University Avenue, Madison, WI 53706, USA; 2 UW Carbone Cancer Center, University of Wisconsin Madison, School of Medicine and Public Health, University of Wisconsin Madison, 600 Highland Avenue, Madison, WI 53792, USA; 3 Molecular and Environmental Toxicology Center, School of Medicine and Public Health, University of Wisconsin Madison, 1300 University Avenue, Madison, WI 53706, USA; 4 Research Animal Resources Center, Graduate School, University of Wisconsin Madison, 1710 University Avenue, Madison, WI 53726, USA; 5 Laboratory for Optical and Computational Instrumentation, Graduate School, University of Wisconsin Madison, 1675 Observatory Dr, Madison, WI 53706, USA

**Keywords:** ACI rat, BN rat, Breast cancer susceptibility, Cell proliferation, Gene expression, Epithelial density

## Abstract

**Background:**

We are using ACI and BN rats, which differ markedly in their susceptibility to 17β-estradiol (E2)-induced mammary cancer, to identify genetic variants and environmental factors that determine mammary cancer susceptibility. The objective of this study was to characterize the cellular and molecular responses to E2 in the mammary glands of ACI and BN rats to identify qualitative and quantitative phenotypes that associate with and/or may confer differences in susceptibility to mammary cancer.

**Methods:**

Female ACI and BN rats were treated with E2 for 1, 3 or 12 weeks. Mammary gland morphology and histology were examined by whole mount and hematoxylin and eosin (H&E) staining. Cell proliferation and epithelial density were evaluated by quantitative immunohistochemistry. Apoptosis was evaluated by quantitative western blotting and flow cytometry. Mammary gland differentiation was examined by immunohistochemistry. Gene expression was evaluated by microarray, qRT-PCR and quantitative western blotting assays. Extracellular matrix (ECM) associated collagen was evaluated by Picrosirius Red staining and Second Harmonic Generation (SHG) microscopy.

**Results:**

The luminal epithelium of ACI rats exhibited a rapid and sustained proliferative response to E2. By contrast, the proliferative response exhibited by the mammary epithelium of BN rats was restrained and transitory. Moreover, the epithelium of BN rats appeared to undergo differentiation in response to E2, as evidenced by production of milk proteins as well as luminal ectasia and associated changes in the ECM. Marked differences in expression of genes that encode proteins with well-defined roles in mammary gland development (*Pgr*, *Wnt4*, *Tnfsf11, Prlr, Stat5a, Areg, Gata3*), differentiation and milk production (*Lcn2*, *Spp1*), regulation of extracellular environment (*Mmp7, Mmp9*), and cell-cell or cell-ECM interactions (*Cd44, Cd24, Cd52*) were observed.

**Conclusions:**

We propose that these cellular and molecular phenotypes are heritable and may underlie, at least in part, the differences in mammary cancer susceptibility exhibited by ACI and BN rats.

## Background

In spite of recent advances in diagnosis and treatment, breast cancer remains the second leading cause of cancer-related death in women in the United States. The existence of multiple subtypes of breast cancer, each with unique clinical and/or molecular characteristics, is now well established [1,2]. Multiple genetic and environmental factors contribute to breast cancer development, and it is becoming increasingly clear that development of each breast cancer subtype is influenced by different sets of factors. Known risk factors include a family history of breast cancer, cumulative exposure to endogenous and exogenous estrogens and breast mammographic density [3-9]. Although several genes have been identified that significantly impact breast cancer risk when mutated or aberrantly expressed, only a small fraction of the overall population risk can be attributed to these genes [10-12]. Similarly, the genetic determinants of responsiveness to estrogens and mammographic density remain poorly defined.

We are using inbred ACI (August x Copenhagen, Irish), COP (Copenhagen) and BN (Brown Norway) rats to define the mechanisms through which estrogens contribute to mammary cancer development and identify genetic determinants of susceptibility to mammary cancer. When treated continuously with 17β-estradiol (E2), female ACI rats develop mammary carcinoma at an incidence approaching 100% [13]. The mammary cancers that develop in E2 treated ACI rats express estrogen receptor-α (ERα) and progesterone receptor (Pgr), are dependent upon E2 for continued growth and survival, and frequently exhibit chromosome copy number changes and instability [14-16]. Development of mammary cancer in E2 treated ACI rats is dramatically inhibited by concurrent treatment with tamoxifen, indicating a requirement for one or more estrogen receptor mediated mechanisms in tumor development [17,18]. Interestingly, tumor development in ACI rats also requires the action of progesterone [13,19]. By contrast, COP and BN rats are resistant to E2-induced mammary cancer [20-22]. Multiple genetic determinants of susceptibility to E2-induced mammary cancer, designated *Emca1* (***E****strogen-induced****m****ammary****ca****ncer 1*) through *Emca9*, have been mapped in crosses between susceptible ACI rats and resistant COP or BN rats [21-24]. Each of the mapped quantitative trait loci (QTL) encompass segments of the rat genome that are orthologous to regions of the human genome linked to breast cancer risk in genome wide association studies (GWAS). Together, these data indicate that the ACI rat model of E2-induced mammary cancer is a physiologically relevant model for studying the molecular etiology of luminal type breast cancers.

The purpose of this study was to define, both qualitatively and quantitatively, the manner in which the mammary glands of susceptible ACI and resistant BN rats respond to E2. Dramatic differences in multiple cellular and molecular responses to E2 were observed when these two inbred rat strains were compared. These differences contributed to and/or were associated with differences in epithelial density, mammary gland differentiation and ECM, as well as differential expression of many genes of known significance to mammary gland development. We propose that the observed differences in responsiveness of the mammary gland to E2 represent phenotypes that underlie the documented strain differences in susceptibility to mammary cancer and may also contribute to and/or serve as biomarkers of breast cancer risk in humans.

## Methods

### Care and treatment of animals

All procedures involving live animals were approved by the Animal Care and Use Committee of the University of Wisconsin-Madison. Female ACI and BN rats were purchased from Harlan Laboratories (Indianapolis, IN). As described previously, Silastic^TM^ tubing implants (Dow Corning, Midland, MI), empty or containing 27.5 mg of E2 (Sigma-Aldrich, St. Louis, MO), were made and placed surgically into the interscapular region of 9 week old rats; these implants release hormone continuously and maintain circulating E2 at levels normally observed in pregnant rats [13,25]. Groups of sham treated (empty implant) control and E2 treated rats were euthanized 1, 3 or 12 weeks later. Each rat was injected with 5-bromo-2′-deoxyuridine (BrdU, Sigma-Aldrich), administered intraperitoneally in phosphate buffered saline (PBS) at 50 mg/kg body weight, four hours before termination of the experiments. Mammary tissues were collected and processed as described below to quantify various cellular and molecular phenotypes.

### Evaluation of mammary gland morphology and histology

Mammary gland whole mounts were generated to evaluate gland morphology. The left inguinal and abdominal mammary glands were collected, stretched flat onto Apex Superior Adhesive Slides (Leica Biosystems, Buffalo Grove, IL), and fixed in 25% glacial acetic acid in ethanol overnight at room temperature. The glands were stained overnight at room temperature in 2 mg/ml carmine (Sigma-Aldrich) and dehydrated in 70%, 95% and 100% ethanol [26]. Finally, the glands were cleared by submersion in xylene, approximately 100 ml per slide, which was changed daily until the epithelial structures could be clearly observed. The whole mounts were photographed using an SZX9 dissecting microscope equipped with a C-7070 digital camera (Olympus, Center Valley, PA).

To evaluate mammary gland histology, the glands were collected and fixed overnight at room temperature in 4% paraformaldehyde. The fixed tissues were then transferred to 70% ethanol, processed and embedded in paraffin. Sections (5.0 microns) were cut, mounted on slides, stained with H&E and evaluated by bright field microscopy. Photomicrographs were obtained using a Zeiss Axio Imager.M2 microscope equipped with an AxioCam HRc digital camera (Carl Zeiss, Thornwood, NY).

### Quantitative immunohistochemistry (IHC)

Paraffin embedded mammary tissues were cut to 5.0 microns, mounted on slides, deparaffinized in xylene and rehydrated stepwise in ethanol at decreasing concentration (100% (twice), 95%, 90%, 80%, 70%, 50%). The tissues were permeabilized in 0.5% Triton X-100 in PBS and antigens were retrieved by boiling in 0.01 M sodium citrate (pH 6.0) for 10 minutes. The sections were then incubated in 10% goat serum (diluted in PBS) for 1 h at room temperature; incubated overnight at 4°C in a primary antibody, diluted as described in Additional file 1: Table S1; rinsed three times for 5 minutes each with 0.1% Tween-20 in PBS (0.1% PBST); incubated with the appropriate secondary antibody (Additional file 1: Table S1) for 1 hour at room temperature; rinsed three times for 5 minutes each in 0.1% PBST; and incubated in Prolong Gold Anti-Fade plus 4′,6-diamidino-2-phenylindole (DAPI, Life Technologies, Carlsbad CA). The stained sections were visualized by fluorescence microscopy using an Axio Imager.M2 microscope equipped with an Apotome structured illumination imaging system and an AxioCam MRm digital camera (Carl Zeiss).

Quantitative IHC was performed using a Vectra™ multispectral fluorescence imaging system running Nuance 3.0.0 imaging software (Caliper Life Sciences, Hopkinton, MA). High-resolution (1,360 × 1,024 pixel), 8-bit grayscale images were acquired automatically every fourth field over the entire tissue section. The nuclear compartment was defined by DAPI (blue). Cytokeratin 5 (K5) and cytokeratin 8 (K8) were visualized using secondary antibodies conjugated with Alexa Fluor 488 (green) and Alexa Fluor 546 (red), respectively. Cells that incorporated BrdU during the S phase of the cell cycle were visualized using a secondary antibody conjugated with Alexa Fluor 647 (yellow). An unstained section of mammary tissue was used to build a spectral library for autofluorescence. Sections stained with a single chromogen were used to build spectral libraries for DAPI, Alexa Fluor 488, Alexa Fluor 546 and Alexa Fluor 647. These libraries allowed the different fluorophores to be distinguished from one other and from autofluorescence without spectral overlap. The inform 1.2 analysis software (Caliper Life Sciences) was trained to distinguish epithelium from non-epithelium and to define subcellular compartments (nucleus, cytoplasm and membrane) and was subsequently used to quantify the fraction of luminal epithelial cells in the S phase of the cell cycle (K8/BrdU double positive) as well as the number of luminal epithelial cells (DAPI/K8 double positive) per field.

### Quantification of apoptosis

Freshly isolated inguinal and abdominal mammary glands were cut into small segments and digested for 6 hours at 37°C in Dulbecco’s modified Eagle’s medium/F12 (DMEM/F12, Life Technologies) supplemented with 5% fetal bovine serum (FBS, Life Technologies), 300 U/ml collagenase (STEMCELL Technologies, Vancouver, BC) and 100 U/ml hyaluronidase (STEMCELL Technologies). The resulting organoids were reduced to single cells by digestion with 0.25% Trypsin (STEMCELL Technologies), 5mg/ml Dispase (STEMCELL Technologies) and 5,000 U/ml DNase I (Roche Applied Science, Indianapolis, IN). The dissociated cells were filtered through 25 μm cell strainers and were stained with Alexa Fluor 647 labeled Annexin V conjugate (Life Technologies) and propidium iodide (PI, Sigma-Aldrich) [27]. The stained cells were analyzed using a FACSCalibur flow cytometer running CellQuestPro version 5.2.1 data acquisition software (BD Biosciences, San Jose, CA). Subsequent data analyses were performed using FlowJo version 9.6 (TreeStar Inc., Ashland, OR).

### Evaluation of gene expression

Gene expression profiles were defined for ACI and BN rats that had been treated with E2 for 12 weeks (n = 5 rats per group) using Affymetrix Rat Genome 230 2.0 GeneChips Arrays (Affymetrix, Santa Clara, CA) as described previously [24]. The primary microarray data have been deposited in Gene Expression Omnibus under accession number GSE49548. Gene ontology enrichment analyses were performed using Ontologizer 2.0 as described previously [24].

Differential expression of selected genes was further evaluated by quantitative real-time PCR (qRT-PCR). Total RNA was isolated from frozen mammary tissue using an Aurum Total RNA Fatty and Fibrous Tissue Kit (Bio-Rad, Hercules, CA). Single-stranded cDNA was synthesized using 1 μg RNA and an iScript cDNA Synthesis Kit (Bio-Rad). qRT-PCR was performed using TaqMan Gene Expression Master Mix (Life Technologies), a CFX96 multicolor real-time PCR detection system (Bio-Rad) and pre-designed TaqMan primers and probes (Integrated DNA Technologies, Coralville, IA): *Pgr* (Rn.PT.53.36352803), *Wnt4* (Rn.PT.53.34796012), *Tnfsf11* (Rn.PT.53.13125438), *Spp1* (Rn.PT.51.10587746.g), *Lcn2* (Rn.PT.51.11294783), *Mmp7* (Rn.PT.51.10499639), *Mmp9* (Rn.PT.53a.7321135), *Lef1* (Rn.PT.51.5354713) and *Actb* (Rn.PT.51.13516462). The PCR program was 95°C for 10 minutes followed by 40 cycles at 95°C for 10 seconds and 60°C for 45 seconds. The data for each gene were analyzed using the ΔΔCq method and CFX Manager Software version 2.1 (Bio-Rad) and are illustrated relative to expression levels of *Actb*.

### Quantitative western blotting

Frozen mammary tissues were homogenized with PowerGen Model 35 Handheld Homogenizer (Thermo Fisher Scientific, Waltham, MA) in lysis buffer containing 25 mM HEPES (pH 7.4), 300 mM NaCl, 1.5 mM MgCl_2_, 1 mM EGTA, 0.2 mM Na_3_VO_4_, 50 mM glycerophosphate, 0.5% Triton X-100 and 1% Halt Proteinase and Phosphatase Inhibitor Cocktail (Thermo Fisher Scientific). The lysates were centrifuged at 12,000g for 30 min, the supernatants were collected and protein concentration was determined using BCA Protein Assay Reagent (Thermo Fisher). Spp1 and Lcn2 were quantified using the Odyssey Imaging System (LI-COR, Lincoln, NE). Briefly, mammary proteins were separated by SDS-PAGE and transferred to Immobilon-FL PVDF membranes (Millipore, Billerica, MA). The blots were incubated with Odyssey blocking buffer (LI-COR) for 1 h at room temperature; incubated overnight at 4°C with primary antibody diluted in Odyssey blocking buffer containing 0.1% Tween-20 as described in Additional file 1: Table S1; washed four times for 5 minutes each with 0.1% PBST; incubated with the appropriate IRDye-conjugated secondary antibody (Additional file 1: Table S1) for 1 h at room temperature in the dark; washed four times for 5 minutes each with 0.1% PBST; imaged and quantified. Cleaved caspase 3, Mmp7 and Mmp9 were quantified using the ChemiDoc XRS + imaging system (Bio-Rad). SDS-PAGE and protein transfer were performed as described above. The blots were then incubated in PBS containing 5% non-fat milk and 0.1% Tween-20 for 1 hour at room temperature; incubated overnight at 4°C in the same buffer containing primary antibody as indicated in Additional file 1: Table S1; washed four times for 5 minutes each with 0.1% PBST; incubated in horseradish peroxidase conjugated secondary antibody for 1 h at room temperature (Additional file 1: Table S1); and washed four times for 5 minutes each with 0.1% PBST. The proteins were visualized using SuperSignal West Dura Chemiluminescent Substrate (Thermo Fisher), imaged and quantified using Image Lab 4.0.1 software (Bio-Rad). All blots were also probed with an antibody to β-actin (Additional file 1: Table S1) and expression of each protein of interest was normalized relative to the amount of β-actin.

### Evaluation of extracellular matrix collagen

Paraffin embedded mammary tissues were sectioned, deparaffinized, rehydrated and stained with Picrosirius Red (Sigma-Aldrich) to visualize ECM collagen; counter stained with Fast Green FCF (Sigma-Aldrich) to visualize non-collagenous cellular and matrix constituents; imaged and photographed using a BX60 epifluroescence microscope equipped with a DP25 digital camera and cellSens digital imaging software (Olympus). Halogen bulb based illumination was used for polarized light and brightfield microscopy. SHG for visualization of collagen was conducted on a custom multiphoton laser scanning microscope [28,29]. All SHG images were collected at a wavelength of 890nm with a 445 nm (20 nm bandpass) filter (Semrock, Rochester, NY).

### Statistical analysis of data

Differences between groups were evaluated using Student’s two-tailed t-test. Significance was established at *p* < 0.05.

## Results

### Rat strain specific effects of 17β-estradiol on mammary gland morphology and histology

**Figure 1:**
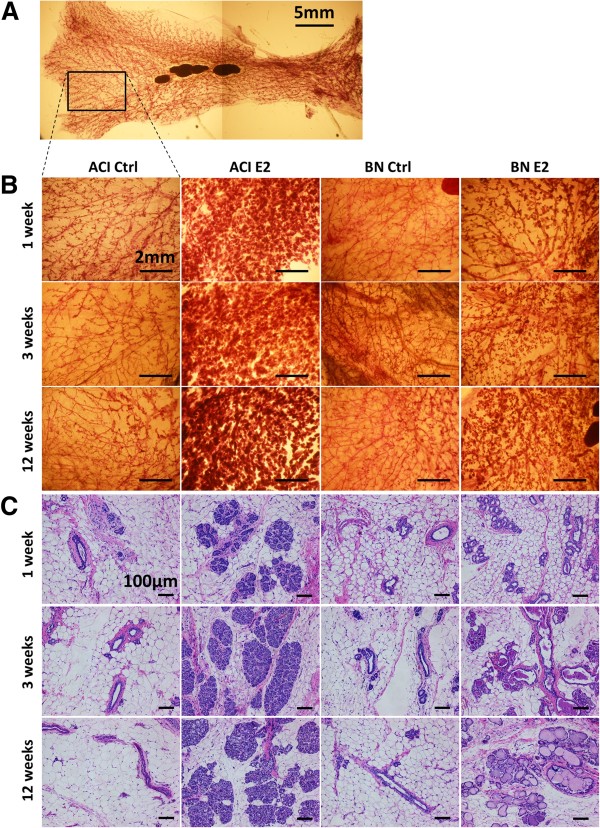
**Rat strain**-**specific effects of 17β**-**estradiol on mammary gland morphology and histology. A,** Representative whole mount of abdominal/inguinal mammary glands from a 10 weeks old ACI female. Left, anterior. Right, posterior. Up, medial. Down, lateral. The rectangle illustrates the approximate region of the abdominal gland illustrated in Panels **B**. Scale bar, 5mm. **B,** Representative images of mammary gland whole mounts from ACI and BN rats, either sham treated (Ctrl) or treated with E2 for 1, 3 or 12 weeks (n = 3). Scale bars, 2mm. **C,** Representative images of mammary tissues, sectioned and stained with hematoxylin and eosin, from ACI and BN rats treated as described in Panels **B** (n ≥ 5). Scale bars, 100 μm.

Mammary gland morphology and histology were evaluated at 1, 3 and 12 weeks relative to the initiation of treatment at 9 weeks of age to determine whether or not the mammary glands of susceptible ACI rats and resistant BN rats differ in their responsiveness to E2. Figure 1A illustrates a typical whole mount of the left abdominal and inguinal mammary glands from a 10 weeks old, ovary intact, ACI rat. Figure 1B represents higher magnification images of the region of the abdominal mammary gland of sham or E2 treated ACI or BN rats represented by the rectangle in Figure 1A. The mammary glands of sham treated ACI and BN rats were comprised of elongated, branched ductal structures that extended to the margins of the mammary fat pad and terminated in small clusters of epithelial cells. No discernible differences in mammary gland morphology were observed between sham treated ACI rats and BN rats. E2 treatment induced a marked increase in the size and complexity of the epithelial structures in the mammary glands of ACI rats. This response was observed within 1 week of initiation of E2 treatment and remained apparent following 3 and 12 weeks of treatment. By contrast, the impact of E2 treatment on the size and complexity of the epithelial structures in BN rats was modest (Figure 1B).

Examination of H&E stained sections demonstrated that the mammary glands of sham treated ACI and BN rats consisted of ducts, terminal duct lobule units and associated ECM embedded within the mammary fat pad (Figure 1C). No discernible differences in mammary gland histology were observed between sham treated ACI and BN rats at any of the three time points. The mammary glands of E2 treated ACI rats consisted of large clusters of epithelial cells organized around the mammary ducts, consistent with induction of lobuloalveolar hyperplasia. This hyperplastic response to E2 was apparent within 1 week of initiation of treatment and appeared similar following 3 and 12 weeks of treatment. Although E2 treatment led to an increase in the apparent size of the epithelial structures in the mammary glands of BN rats, this resulted primarily from luminal ectasia in addition to a slight but discernible induction of lobuloalveolar hyperplasia. The luminal ectasia was apparent within 1 week of initiation of E2 treatment and remained the predominant feature in the mammary glands of E2 treated BN rats following 3 and 12 weeks of treatment. Together, these data illustrate remarkable differences in the cellular responses to E2 within the mammary glands of ACI and BN rats that are discernible within 1 week of initiation of hormone treatment.

### Rat strain specific effects of 17β-estradiol on mammary cell proliferation and differentiation, but not apoptosis

**Figure 2:**
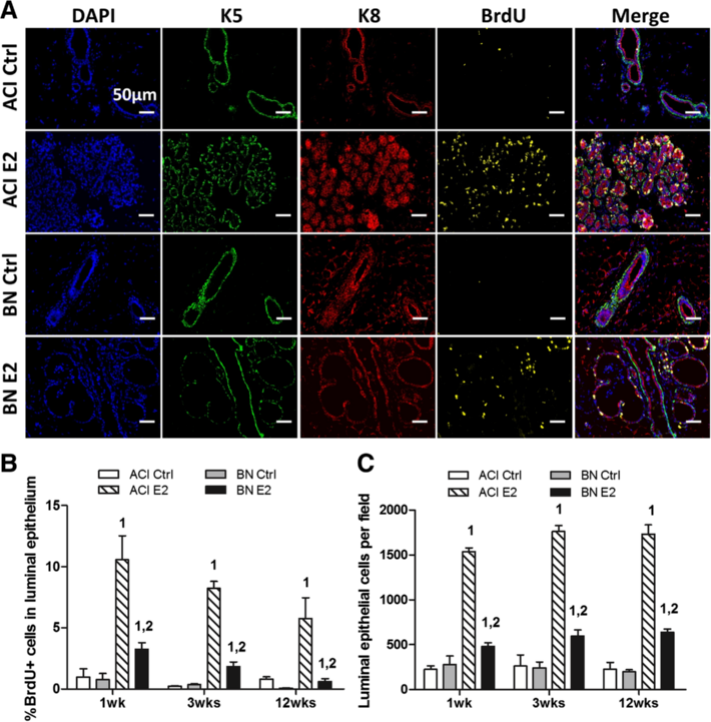
**Rat strain**-**specific effects of 17β**-**estradiol on mammary epithelial cell proliferation. A,** Representative fluorescent images of mammary tissues from ACI and BN rats, either sham treated (Ctrl) or treated with E2 for 1 week (n = 3). Column 1, nuclei identified by staining DNA with 4′,6-diamidino-2-phenylindole (DAPI, blue). Column 2, basal epithelial cells were identified by immunostaining for cytokeratin 5 (K5, green). Column 3, luminal epithelial cells were identified by immunostaining for cytokeratin 8 (K8, red). Colum 4, cells transiting S phase were identified by immunostaining for BrdU (yellow). Column 5, merged images from columns 1 through 4. Scale bars, 50 μm. **B,** The number of luminal epithelial cells (K8 positive) in S phase (BrdU positive) was quantified using a Vectra^TM^ multispectral fluorescence imaging system and illustrated as the percentage of total luminal epithelial cells. **C,** The number of luminal epithelial cells per field was quantified as an indicator of epithelial density. Each data bar in Panels B and C represents the mean ± standard error of the mean (SEM, n = 3). 1, *p* < 0.05 for comparison of E2 treated vs. sham treated rats of same strain. 2, *p* < 0.05 for comparison of E2 treated BN vs. treated ACI rats.

Proliferation in defined mammary cell populations was quantified by IHC using antibodies to K5, a marker of basal epithelium, K8, a marker of luminal epithelium, and BrdU, a marker for cells that transited the S phase of the cell cycle within the 4 hours preceding euthanasia. Representative images from ACI and BN rats treated for 1 week with E2 and age matched, sham treated, control rats are illustrated in Figure 2A. Images generated at the 3 week and 12 week time points are appended as Additional file 2: Figure S1A and S1B, respectively. The mammary epithelia of both control and E2 treated ACI and BN rats were comprised of an outer layer of basal cells surrounding the inner luminal cells. Quantification by Vectra system demonstrated that the fraction of BrdU positive cells in the luminal epithelium of sham treated ACI and BN rats was below 1.0% at each of the time points and did not differ between strains (Figure 2A and 2B). Treatment with E2 dramatically induced proliferation within the luminal epithelium of ACI rats. The fraction of luminal cells staining positive for BrdU was increased to 10.6%, 8.2% and 5.8% in ACI rats treated with E2 for 1, 3 and 12 weeks, respectively. By contrast, E2 treatment increased the fraction of luminal cells staining positive for BrdU in BN rats to only 3.2% following 1 week and 1.8% following 3 weeks of treatment, and no significant increase was observed in BN rats treated with E2 for 12 weeks (Figure 2A and 2B). The fraction of S phase cells in the luminal epithelium of E2 treated ACI rats was significantly greater than in treated BN rats at each of the three time points. The difference in induction of luminal epithelial cell proliferation in these two rat strains was clearly reflected in the morphological and histological differences described above (Figure 1B, 1C and 2A), as well as in differences in epithelial density measured by quantifying the number of luminal epithelial cells per microscopic field. This indicator of epithelial density did not differ between sham treated ACI and BN rats at any of the time points examined (Figure 2C). The number of luminal epithelial cells per field was increased more than 6-fold in ACI rats treated with E2 for 1, 3 or 12 weeks, relative to age-matched control ACI rats. By contrast, the number of luminal epithelial cells per field was increased 1.7-, 2.4- and 3.2-fold in BN rats treated for 1, 3 and 12 weeks, respectively, relative to control BN rats. Together, these data demonstrate that the proliferative response of the luminal epithelium of ACI rats to E2 is markedly greater than that of BN rats. Proliferation in the basal epithelium was not quantified because the basal cells in E2 treated rats assumed an elongated morphology that made it difficult to assign a specific nucleus to the cells staining positive for K5.

**Figure 3:**
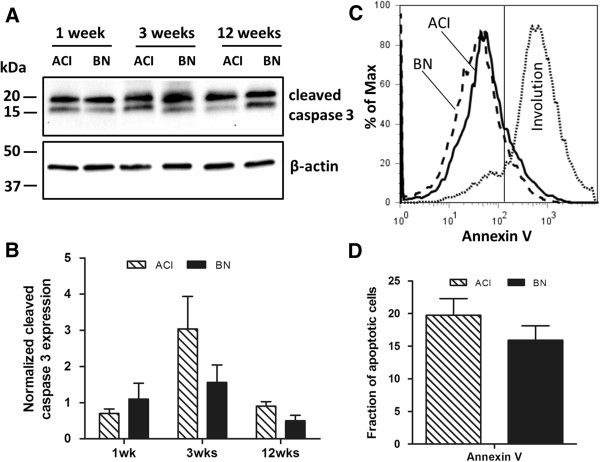
**No discernable effect of rat strain on apoptosis in the mammary gland. A,** Representative western blots of protein lysates prepared from mammary glands of ACI and BN rats, treated with E2 for 1, 3 or 12 weeks, and probed with antibody to cleaved forms (19 kDa and 17 kDa fragments) of caspase 3 (n = 3). **B,** The amount of cleaved caspase 3 was quantified using a Bio-Rad ChemiDoc XRS + imaging system and normalized to the amount of β-actin. Each data bar represents the mean ± SEM, n = 3 biological replicates. **C,** Cells isolated from mammary glands of ACI and BN rats, treated with E2 for 3 weeks, were stained with Annexin V and propidium iodide and analyzed by flow cytometry. Cells isolated from an involuting mammary gland (3 days post-lactation) were stained and analyzed as a positive control. **D,** Each data bar represents the number of apoptotic cells (positive for annexin V, negative for PI) expressed as a percentage of total PI negative cells (mean ± SEM, n = 3).

Apoptosis within the mammary gland was evaluated using two independent methods. In the first, the levels of the activated 17 and 19 kDa forms of caspase 3 were quantified by western blotting. No significant differences in the levels of cleaved caspase 3 were observed when mammary glands from E2 treated ACI and BN rats were compared (Figure 3A and 3B). Binding of Annexin V to dispersed mammary cells was quantified by flow cytometry as a second indicator of apoptosis. Approximately 20% of cells isolated from mammary glands of ACI and BN rats that were treated with E2 for 3 weeks stained positive for Annexin V and negative for PI (Figure 3C and 3D). When an involuting mammary gland from an ACI rat (3 days post-lactation) was evaluated as a positive control, approximately 80% of cells isolated cells stained positive for Annexin V. Together, these data suggest that the levels of apoptosis in the mammary glands of E2 treated ACI and BN rats did not differ significantly.

**Figure 4:**
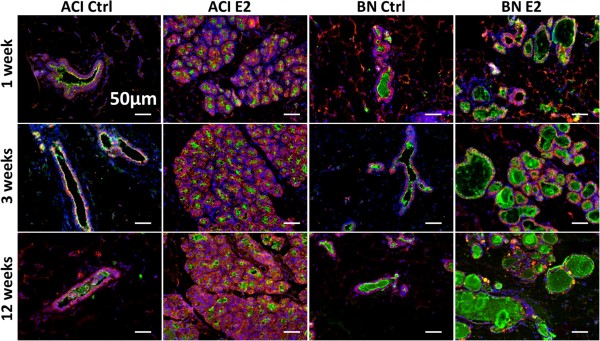
**Rat strain**-**specific effects of 17β**-**estradiol on luminal ectasia and expression of milk proteins.** Representative fluorescent images of mammary tissues from ACI and BN rats, either sham treated (Ctrl) or treated with E2 for 1, 3 or 12 weeks (n = 3). Nuclei were identified by staining DNA with DAPI (blue), luminal epithelial cells by immunostaining for K8 (red), and milk proteins were identified by immunostaining using a polyclonal antibody generated against milk specific proteins (green). Note that the mammary glands from E2 treated BN rats exhibit prominent ectatic lumens containing immunoreactive milk protein(s). Scale bars, 50 μm.

IHC was performed using an antibody to milk proteins to evaluate mammary gland differentiation and to define the nature of the luminal ectasia observed in E2 treated BN rats (Figure 4). Immunoreactive milk proteins were detected in the lumens of sham treated ACI and BN rats and the amount of immunostaining did not differ discernibly between these rat strains. Milk proteins were also detected in the lumens of ACI rats treated with E2 for 1, 3 and 12 weeks. The most prominent feature of the mammary glands of E2 treated BN rats was the markedly dilated lumens that contain immunoreactive milk proteins. These data, together with data presented above, suggest that the primary response of the ACI mammary gland to E2 is cell proliferation, which leads to dramatic epithelial hyperplasia. By contrast, the primary response of the BN mammary gland to E2 appears to be differentiation to an active secretory epithelium associated with luminal ectasia and modest epithelial hyperplasia.

### Rat strain specific effects of 17β-estradiol on gene expression

To gain insights into the molecular mechanisms that underlie the observed differences in responsiveness of the ACI and BN mammary glands to estrogen, gene expression profiles were generated using total RNA isolated from whole mammary glands from ACI and BN rats that were treated with E2 for 12 weeks. Transcripts corresponding to 4170 probe sets were observed to be differentially expressed using a false discovery rate of 5%. Of these, transcripts corresponding to 2267 probe sets were more highly expressed in mammary glands from E2 treated ACI rats, relative to matched BN rats, whereas transcripts corresponding to 1903 probe sets were more highly expressed in mammary glands from BN rats (Additional file 1: Table S2). The genome ontology terms most strongly associated with the differentially expressed transcripts related to immune system process/response, cell activation/proliferation and cell surface binding/adhesion (Additional file 1: Table S3).

**Figure 5:**
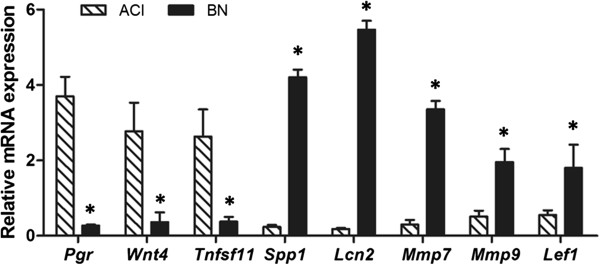
**Rat strain**-**specific effects of 17β**-**estradiol on mRNA expression.** RNA was isolated from mammary glands of ACI and BN rats, treated with E2 for 12 weeks. Expression of mRNAs encoded by 8 genes of interest was quantified using the ΔΔCq PCR-based method. Each data bar represents the level of the indicated mRNA normalized to the level of β-actin mRNA (mean ± SEM, n = 3. *, *p* < 0.05). The differences observed upon comparison of expression of these 8 mRNA in mammary glands from E2 treated ACI and BN rats were concordant with differences in expression observed upon microarray analysis.

Several genes that encode proteins that serve defined roles in mammary gland development were observed to be more highly expressed in mammary glands of E2 treated ACI rats, including *Pgr*, *Wnt4*, *Tnfsf11*, *Areg*, *Prlr*, *Stat5a* and *Gata3*. Interestingly, two genes that encode proteins that are secreted into milk and may function in regulation of mammary gland differentiation and milk production, *Spp1* and *Lcn2*, were more highly expressed in the mammary glands of BN rats. Also highly expressed in the mammary glands of E2 treated BN rats were multiple genes that encode proteins that regulate the extracellular environment including *Mmp7*, *Mmp9*, *Mmp11* and *Mmp12*; *Adam8*, *Adam9*, *Adam15* and *Adam17*; and *Timp1*, *Timp2* and *Timp3*. qRT-PCR was performed for eight differentially expressed genes to validate the microarray data. The data from these analyses verified that *Pgr, Wnt4* and *Tnfsf11* were expressed at a significantly higher level in the mammary glands of E2 treated ACI rats, whereas *Spp1, Lcn2, Mmp7, Mmp9, and Lef1* were expressed at a significantly higher level in the mammary glands of E2 treated BN rats (Figure 5).

**Figure 6:**
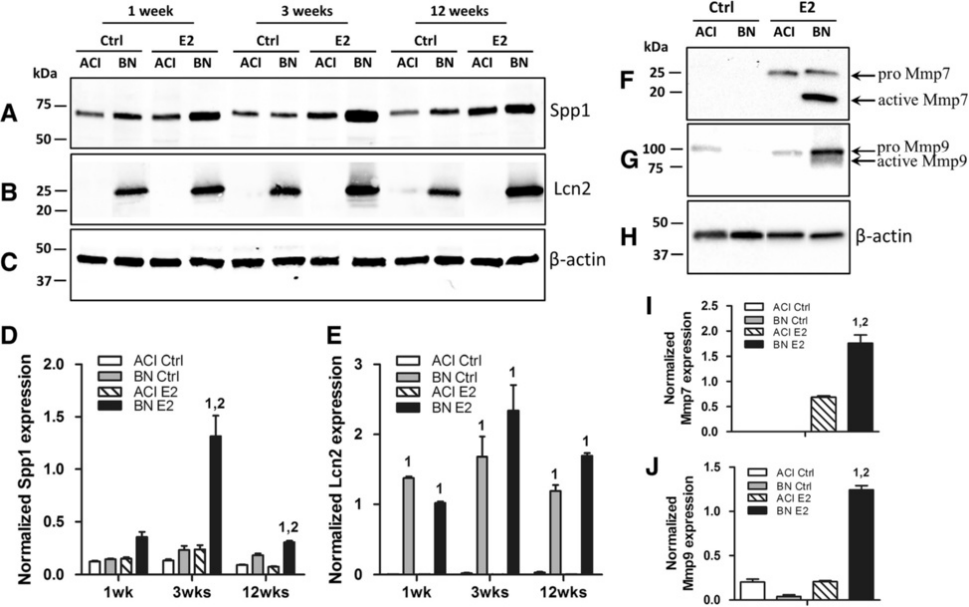
**Rat strain**-**specific effects of 17β**-**estradiol on protein expression.** Representative western blots of protein lysates prepared from mammary glands of ACI and BN rats, treated with E2 for 1, 3 or 12 weeks, and probed with antibody to Spp1 **(Panel A)**, Lcn2 **(Panel B)** or β-actin **(Panel C)**. The amounts of Spp1 **(Panel D)** and Lcn2 **(Panel E)** were quantified using a LI-COR Odyssey system and expressed relative to the amount of β-actin in the same lysate. Representative western blots of protein lysates prepared from mammary glands of ACI and BN rats, treated with E2 for 12 weeks, and probed with antibody to Mmp7 **(Panel F)**, Mmp9 **(Panel G)** or β-actin **(Panel H)**. The amounts of the proenzymes and active forms of Mmp7 **(Panel I)** and Mmp9 **(Panel J)** were quantified using a Bio-Rad ChemiDoc XRS + imaging system and expressed relative to the amount of β-actin in the same lysate. Each data bar represents the mean ± SEM, n = 3 biological replicates. 1, *p* < 0.05, E2 treated vs. sham treated rats of same strain. 2, *p* < 0.05, E2 treated BN rats compared to E2 treated ACI rats.

Expression of a subset of the genes that are potentially of functional significance in relation to mammary development, ECM and/or ECM remodeling and mammary cancer susceptibility was further evaluated at the protein level. Although Spp1 was expressed at similar levels in control ACI and BN rats, expression increased in response to E2 treatment in mammary glands of BN but not ACI rats, resulting in significantly higher levels of Spp1 in treated BN rats at the 3 (5.5-fold) and 12 (4.1-fold) week time points, relative to treated ACI rats (Figure 6A and 6D). Lcn2 was virtually undetectable in mammary glands of control and E2 treated ACI rats. By contrast, Lcn2 was highly expressed in mammary glands of control and E2 treated BN rats (Figure 6B and 6E). Mmp7 was undetectable in mammary glands of control ACI and BN rats at each of the three time points examined (data not shown), remained undetectable in the mammary glands of ACI and BN rats treated with E2 for 1 week, but was detected in glands from ACI and BN rats treated with E2 for 3 (data not shown) and 12 weeks (Figure 6F and 6I). Moreover, the active 18kDa form of Mmp7 predominated over the 25kDa proenzyme in mammary glands from BN rats treated with E2 for 12 weeks (Figure 6F and 6I). Mmp9 was expressed at similar levels in mammary glands of control and E2 treated ACI and BN rats at the 1 and 3 week time points (data not shown). At the 12 week time point, Mmp9 was expressed at a higher level in E2 treated BN rats, relative to treated ACI rats, and the active form of Mmp9 was observed only in mammary glands from the treated BN rats (Figure 6G and 6J).

### Rat strain specific effects of 17β-estradiol on extracellular matrix

**Figure 7:**
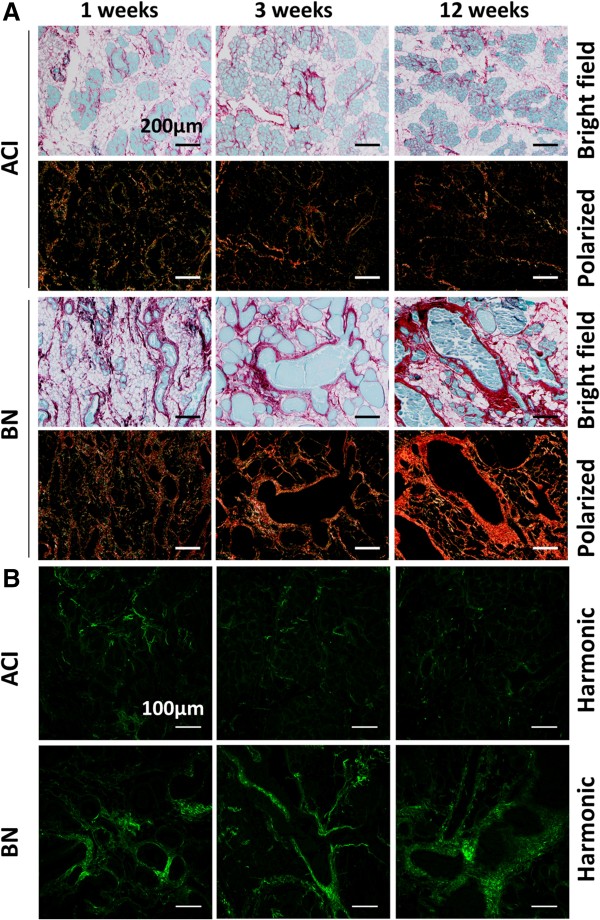
**Rat strain**-**specific effects of 17β**-**estradiol on extracellular matrix and associated collagens. A,** Representative images of mammary tissues, sectioned and stained with Picrosirius Red, from ACI and BN rats treated with E2 for 1, 3 or 12 weeks (n = 3). When imaged under bright field illumination, ECM associated collagens appear red, non-collagenous tissue and intraluminal secreta appear green. When imaged under polarized light, collagens appear green-yellow-orange-red. Scale bars, 200 μm. **B,** Representative SHG images of mammary tissues from ACI and BN rats treated with E2 for 1, 3 or 12 weeks (n = 3). Scale bars, 100 μm. Together, these images illustrate a robust collagenous stroma associated with ectatic ducts in mammary glands of E2 treated BN rats.

Mammary tissues from ACI and BN rats were stained with Picrosirius Red and examined using histopathology and SHG imaging methods to evaluate ECM and associated collagens. When examined using bright field microscopy, all collagen types appear red, while non-collagenous tissues and intraluminal secreta appear green. Under polarized light, the collagen fibers are birefringent in a range of colors from green-yellow-orange-red. When evaluated qualitatively, mammary tissues from sham treated ACI and BN rats did not differ discernibly with respect to the mammary parenchyma, stroma, ECM or collagen (data not shown). By contrast, the ectatic ducts uniquely present in the mammary glands of E2 treated BN rats were generally associated with a robust collagenous stroma (Figure 7A). Evaluation of the ECM using SHG further established the existence of a robust collagenous stroma surrounding ectatic ductal structures in E2 treated BN rats. However, the morphology and content of stromal collagen appeared to be qualitatively and anatomically appropriate to the size of the ducts present (Figure 7B).

## Discussion

Data presented herein demonstrate that the mammary glands of ACI and BN rats exhibited marked quantitative and qualitative differences in their cellular and molecular responses to E2. The primary response exhibited by ACI rats, which are uniquely susceptible to mammary cancer when treated with estrogens, was a robust and sustained proliferation within the mammary epithelium. By contrast, the proliferative response of the mammary epithelium of BN rats, which are highly resistant to estrogen-induced mammary cancer, was restrained and transient. This difference in induction of cell proliferation, not a difference in apoptosis, appeared to be largely responsible for the quantitative differences in epithelial density observed when the mammary glands of ACI and BN rats were compared following 1, 3 and 12 weeks of E2 treatment. Moreover, the mammary glands of E2 treated BN rats, but not ACI rats, exhibited qualitative phenotypes consistent with differentiation to secretory epithelium, as well as luminal ectasia and associated changes in collagenous stroma. These differences in the responsiveness of the mammary glands of ACI and BN rats to E2 were apparent within one week of initiation of treatment, strongly suggesting that the molecular mechanisms responsible for the rat strain specific responses may be inherent within the mammary glands of these inbred rat strains.

Comparison of gene expression profiles for mammary glands of E2 treated ACI and BN rats revealed differential expression of multiple genes that may have contributed to the differences in luminal epithelial cell proliferation and lobuloalveolar hyperplasia observed upon comparison of these rat strains. *Pgr*, *Wnt4*, *Tnfsf11* (*RankL*), *Prlr*, *Stat5a*, *Areg* and *Gata3* were expressed at higher levels in mammary glands of E2 treated ACI rats, relative to identically treated BN rats. The protein products encoded by these genes play well defined important roles in mammary gland development. Expression of *Pgr* in mammary epithelium is induced by E2 and progesterone, acting through Pgr, plays a requisite role in stimulating lobuloalveolar development during pregnancy [30-32]. Moreover, studies summarized above have demonstrated a requisite role for progesterone in the induction of mammary cancer development by E2 in ACI rats [13,19]. Both Wnt4 and RankL have been demonstrated to function downstream of Pgr in stimulating lobuloalveolar development and have more recently been shown to be requisite paracrine mediators of the actions of progesterone in the regulation of mammary stem cell number [33-37]. Prlr and Stat5a are both required for induction of lobuloalveolar development by prolactin, a second major hormonal regulator of lobulogenesis during pregnancy [38,39]. Areg functions as an important paracrine mediator of the actions of estrogens and ERα on induction of mitogenesis in the mammary epithelium [40,41]. Finally, Gata3 is required for elongation of mammary ducts at puberty and maintenance of differentiated luminal epithelium, and also acts as a positive regulator of expression of *Esr1*, the gene encoding ERα [42,43]. Additional studies are needed to establish whether differential expression of these genes is the cause or the consequence of the observed differences in epithelial cell proliferation and lobuloalveolar hyperplasia exhibited by E2 treated ACI and BN rats.

Other differentially expressed genes encode protein products that are functionally associated with mammary gland differentiation, lactation and/or post-lactational involution. *Spp1* and *Lcn2* are among those genes that were most highly expressed at the mRNA level in mammary glands of E2 treated BN rats, relative to identically treated ACI rats. *Spp1* encodes a secreted phosphoprotein that is highly expressed in the mammary gland during lactation and involution [44-46]. *Spp1* has also been demonstrated to be more highly expressed in mammary glands of parous mice and rats, compared to nulliparous controls [47,48]. Inhibition of *Spp1* expression in the luminal epithelium of the mouse mammary gland inhibits lobuloalveolar development, expression of genes encoding milk proteins and milk production [49]. Moreover, *Spp1* underlies a quantitative trait locus (QTL) in dairy cattle that controls milk yield and protein content [45]. Together, these data suggest that Spp1 regulates multiple processes in the mammary epithelium during pregnancy, lactation and/or mammary gland involution. *Lcn2* encodes a secreted glycoprotein that is highly expressed within the luminal epithelium of the mammary gland during pregnancy and lactation as well as during mammary gland involution [50,51]. Lcn2 is known to bind a diverse group of ligands, including retinoids, fatty acids, bacterial siderophores and specific MMPs. Suggested functions of Lcn2 in the mammary gland include modulation of inflammation and immunity, ECM remodeling and regulation of iron homeostasis. The functional significance of differential expression of *Spp1* and *Lcn2* in the mammary glands of ACI and BN rats remains under investigation.

As noted above, luminal ectasia and associated collagenous stroma were qualitative phenotypes unique to the mammary glands of E2 treated BN rats. Several genes that encode proteins that are known to modify the extracellular microenvironment were observed to be differentially expressed between E2 treated ACI and BN rats. Two examples are *Mmp7* and *Mmp9*, both of which were expressed and activated to a greater degree in the mammary glands of BN rats, relative to ACI rats. Known functions of these MMPs include ECM remodeling and production of active forms of multiple growth factors, cytokines and chemokines [52]. *Mmp7* is unique among the MMPs in that its expression in the mammary gland is largely restricted to the glandular epithelium [53]. A role for Mmp7 in mammary gland development is suggested by a study that demonstrated that expression of MMP7 in the mammary epithelium of nulliparous mice under control of the mouse mammary tumor virus (MMTV) promoter induced production of milk proteins, suggesting MMP7 may play a role in mammary gland differentiation [54]. Other studies support a role of Mmp7 in mammary cancer development and/or progression. For example, expression of an *MMTV-MMP7* transgene in the mammary epithelium resulted in development of hyperplastic alveolar nodules in a large fraction of aged multiparous mice and shortened the time to onset of mammary tumors in mice that also expressed an *MMTV-Neu* transgene [53]. Moreover, single nucleotide polymorphisms in *MMP7* have been associated with disease free and/or overall survival in two breast cancer case control studies [55,56]. Mmp9 is expressed by the mammary epithelium, stromal fibroblasts and infiltrating immune/inflammatory cells. The highest levels of Mmp9 expression in the mammary gland occur during pregnancy and involution [57,58]. However, the roles of Mmp9 at these developmental stages are not well defined. Mmp9 contributes to mammary cancer metastasis in mouse models and nucleotide variants within *MMP9* have been associated with breast cancer metastasis in humans [59,60]. Interestingly, Mmp9 has been demonstrated to form a binary complex with Lcn2, leading to activation and stabilization of this matrix metalloproteinase [61-64]. These data suggest a potential mechanism for the enhanced activation of Mmp9 observed in the mammary glands of E2 treated BN rats.

Comparison of gene expression profiles for mammary glands from E2 treated ACI and BN rats also revealed differential expression of many genes that encode proteins that reside on the cell surface and function in cell-cell or cell-ECM interactions. One such gene, *Cd44*, was observed to be expressed at an approximate 10-fold higher level in BN rats than in ACI rats. Cd44 is expressed by the myoepithelium in developing mammary gland and by luminal epithelium in adult mouse mammary gland and human breast [65]. *Cd44* null mice exhibit a lactation defect which appears to result from reduced activation of heparin binding epidermal growth factor (HB-EGF) and downstream signaling through ErbB4 [66]. *Cd44* null mice also exhibit delayed ductal outgrowth and small TEBs and these phenotypes were attributed to aberrant interactions between myoepithelium and luminal epithelium [65]. Multiple studies have demonstrated a physical interaction between CD44 and Spp1 in a wide variety of cell types, including breast cancer cells, which alters an array of cellular phenotypes including motility and invasiveness [67-70]. CD44 has also been demonstrated to interact physically and functionally with Mmp7 and Mmp9 in multiple cell types, and by doing so enhances the activities of Mmp7 and Mmp9 on specific substrates within the extracellular environment [66,71-73]. Interestingly, the interaction between CD44 and Mmp9 in PC3 prostate cancer cells has been demonstrated to be induced by Spp1 [74]. CD24 and CD52 were observed to be expressed at higher levels in mammary glands from E2 treated ACI rats, relative to BN rats. *CD24* encodes a cell surface glycoprotein that has emerged as a marker for mammary stem cells [75,76]. In the mouse mammary gland, Cd24 is expressed in the luminal epithelium and to a lesser extent in the basal epithelium [77,78]. Mice that are homozygous for a *Cd24* null allele exhibit accelerated ductal elongation and increased branching morphogenesis in the mammary gland [78]. *CD52*, which is paralagous to *CD24*, is expressed by lymphocytes and other types of immune cells. Virtually nothing is known regarding the role of CD52 in mammary gland development or function. Ongoing studies are focused on identifying and quantifying the cell types in the mammary glands of ACI and BN rats that express these different proteins.

We hypothesize that variation in a subset of the cellular and molecular phenotypes described herein is heritable and underlies the differing susceptibilities of the ACI and BN rats to E2-induced mammary cancer. We are currently testing this hypothesis by evaluating these phenotypes in a panel of unique congenic rat strains that were developed to characterize the QTL that were identified as genetic determinants of susceptibility to E2-induced mammary cancer in intercrosses between susceptible ACI and resistant BN rats [22,23]. Our working model is that genetic variants within the *Emca* QTL impact expression of genes that function downstream of E2 and progesterone to control proliferation, survival and/or differentiation within the mammary epithelium and/or the cellular composition of the stroma and thereby influence susceptibility to E2-induced mammary cancer. Supporting this model is a recently published study in which it was demonstrated that congenic rats that harbor, on the ACI genetic background, BN alleles across the *Emca8* locus on rat chromosome 5 exhibited significantly reduced susceptibility to E2-induced mammary cancer that was accompanied by reduced expression in the mammary gland of *Pgr*, *Wnt4* and *Cd52* and increased expression of *Spp1*, relative to E2 treated ACI rats [24]. We further hypothesize that variation in the different cellular and molecular phenotypes observed in E2 treated ACI and BN rats is representative of variation that would exist within the genetically heterogeneous human population. For example, the difference in mammary epithelial density exhibited by E2 treated ACI and BN rat may be analogous to variation in breast mammographic density in humans, which is known to be modified by estrogens as well as other hormonal, genetic and environmental factors and has been strongly associated with breast cancer risk. Additional studies are required to establish cause and effect relationships between the cellular, molecular and mammary cancer susceptibility phenotypes in the rat and to translate the knowledge gained to humans.

## Conclusions

The mammary glands of susceptible ACI and resistant BN rats exhibited marked quantitative and qualitative differences in their cellular and molecular responses to E2.

The luminal epithelium of ACI rats exhibited a rapid and sustained proliferative response to E2, resulting in lobuloalveolar hyperplasia and increased epithelial density. By contrast, the epithelium of BN rats exhibited responses indicative of differentiation to secretory epithelium, as well as luminal ectasia and associated changes in the ECM. Comparison of gene expression profiles for mammary glands of E2 treated ACI and BN rats revealed differences in expression of multiple genes whose protein products are required for normal mammary gland development, differentiation and milk production, regulation of the extracellular environment, and cell-cell or cell-ECM interactions. Ongoing studies are focused on defining the relationships between these cellular and molecular phenotypes and the genetically determined differences in susceptibility of ACI and BN rats to E2-induced mammary cancer.

## Abbreviations

ACI: August x Copenhagen, Irish; COP: Copenhagen; BN: Brown Norway; E2: 17β-estradiol; ERα: Estrogen receptor-α; Pgr: Progesterone receptor; Emca: ***E****strogen-induced ****m****ammary ****ca****ncer*; QTL: Quantitative trait loci; GWAS: Genome wide association studies; ECM: Extracellular matrix; PBS: Phosphate buffered saline; DAPI: 4′,6-diamidino-2-phenylindole; BrdU: 5-bromo-2′-deoxyuridine; H&E: Hematoxylin and eosin; K5: Cytokeratin 5; K8: Cytokeratin 8; DMEM: Dulbecco’s modified Eagle’s medium; PI: Propidium iodide; qRT-PCR: Quantitative real-time PCR; IHC: Immunohistochemistry; SHG: Second Harmonic Generation.

## Competing interests

The authors declare that they have no competing interests.

## Authors’ contributions

Conception and design, LD, JDS; development of methodology, LD, JDS; acquisition of data, LD, YZ, CLW, RS, KWE, JDS; analysis and interpretation of data, LD, YZ, CLW, RS, KWE, JDS; writing of the manuscript: LD, RS, JDS; study supervision: JDS. All authors read and approved the final manuscript.

## Pre-publication history

The pre-publication history for this paper can be accessed here:

http://www.biomedcentral.com/1471-2407/13/573/prepub

## Supplementary Material

Additional file 1 **Table S1.** Primary and secondary antibodies for immunohistochemistry and western blot; **Table S2**: Rat strain specific effects of 17β-Estradiol on gene expression; **Table S3**: Gene ontology analysis with the differentially expressed transcripts. Click here for file

Additional file 2 **Figure S1.** Rat strain-specific effects of 17β-estradiol on mammary epithelial cell proliferation. Click here for file
