# Supplementary material for: Association of cellular and molecular responses in the rat mammary gland to 17β-estradiol with susceptibility to mammary cancer
Source: BMC Cancer. 2013 Dec 5;13:573. doi: 10.1186/1471-2407-13-573 (PMC3924185; doi:10.1186/1471-2407-13-573)
Supplement: Additional file 2 — Figure S1. Rat strain-specific effects of 17β-estradiol on mammary epithelial cell proliferation. [file 1471-2407-13-573-S2.docx]

**Supplementary Figure S1**

**
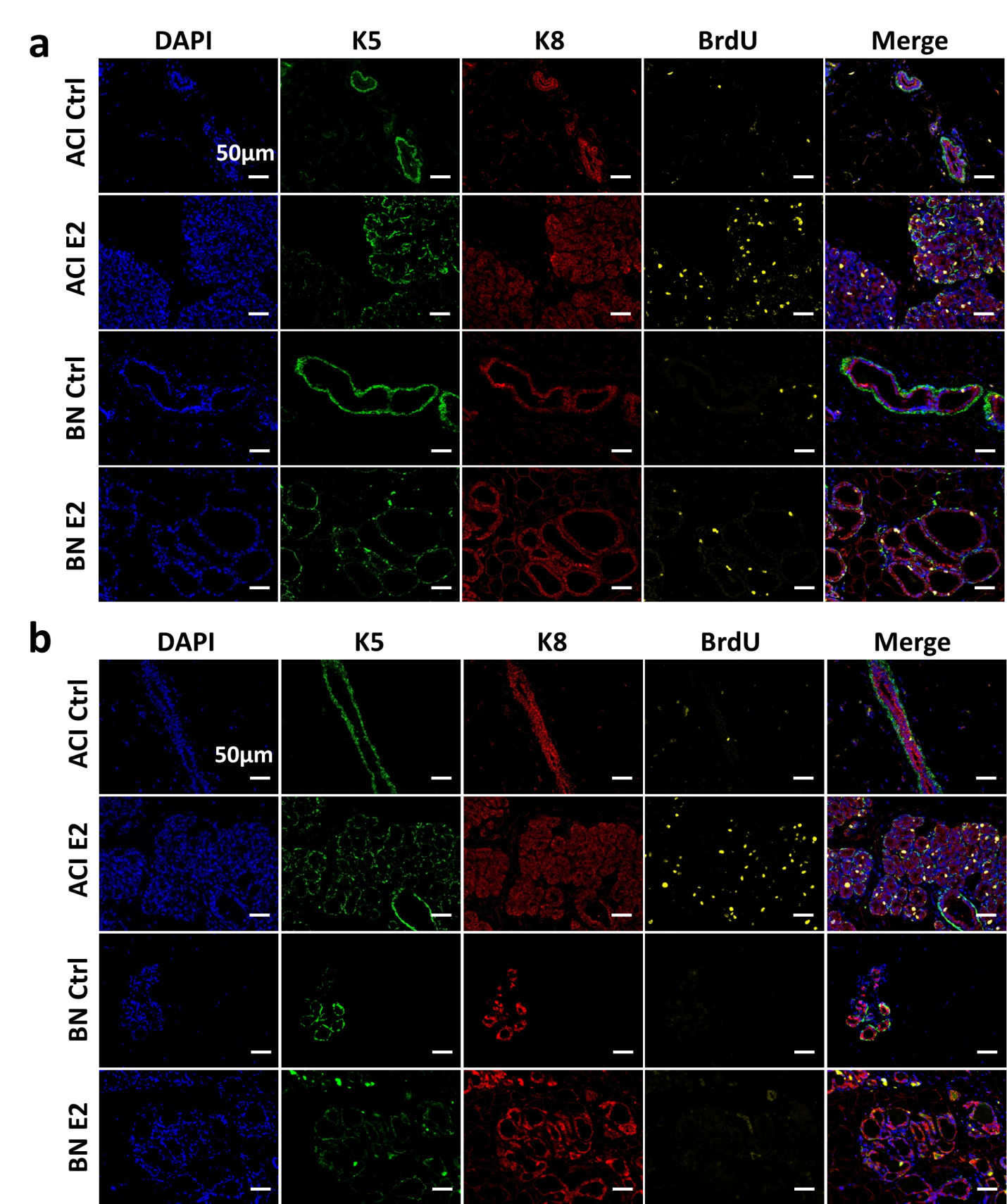
**

**Supplementary Fig. S1.** Rat strain-specific effects of 17β-estradiol on mammary epithelial cell proliferation. A, Representative fluorescent images of mammary tissues from ACI and BN rats, either sham treated (Ctrl) or treated with E2 for 3 weeks (Panels A) or 12 weeks (Panels B). Column 1, nuclei identified by staining DNA with DAPI (blue). Column 2, basal epithelial cells were identified by immunostaining for K5 (green). Column 3, luminal epithelial cells were identified by immunostaining for K8 (red). Colum 4, cells transiting S phase were identified by immunostaining for BrdU (yellow). Column 5, merged images from columns 1 through 4. Scale bars, 50 µm. The number of luminal epithelial cells (K8 positive) and the number of luminal epithelial cells in S phase (BrdU positive) were quantified using a Vectra^TM^ multispectral fluorescence imaging system and the resulting data were illustrated in Figure 2.
